# GmSALT3, Which Confers Improved Soybean Salt Tolerance in the Field, Increases Leaf Cl^-^ Exclusion Prior to Na^+^ Exclusion But Does Not Improve Early Vigor under Salinity

**DOI:** 10.3389/fpls.2016.01485

**Published:** 2016-09-30

**Authors:** Ying Liu, Lili Yu, Yue Qu, Jingjing Chen, Xiexiang Liu, Huilong Hong, Zhangxiong Liu, Ruzhen Chang, Matthew Gilliham, Lijuan Qiu, Rongxia Guan

**Affiliations:** ^1^The National Key Facility for Crop Gene Resources and Genetic Improvement, Institute of Crop Science, Chinese Academy of Agricultural SciencesBeijing, China; ^2^Australian Research Council Centre of Excellence in Plant Energy Biology, School of Agriculture, Food and Wine and Waite Research Institute, University of AdelaideGlen Osmond, SA, Australia

**Keywords:** soybean, salt tolerance, near isogenic line, sodium, chloride, *GmSALT3*, salt exclusion

## Abstract

Soil salinity reduces soybean growth and yield. The recently identified *GmSALT3* (*Glycine max* salt Tolerance-associated gene on chromosome 3) has the potential to improve soybean yields in salinized conditions. Here we evaluate the impact of *GmSALT3* on soybean performance under saline or non-saline conditions. Three sets of near isogenic lines (NILs), with genetic similarity of 95.6–99.3% between each pair of NIL-T and NIL-S, were generated from a cross between two varieties 85–140 (salt-sensitive, S) and Tiefeng 8 (salt-tolerant, T) by using marker-assisted selection. Each NIL-T; 782-T, 820-T and 860-T, contained a common ~1000 kb fragment on chromosome 3 where *GmSALT3* was located. We show that *GmSALT3* does not contribute to an improvement in seedling emergence rate or early vigor under salt stress. However, when 12-day-old seedlings were exposed to NaCl stress, the NIL-T lines accumulated significantly less leaf Na^+^ compared with their corresponding NIL-S, while no significant difference of K^+^ concentration was observed between NIL-T and NIL-S; the magnitude of Na^+^ accumulation within each NIL-T set was influenced by the different genetic backgrounds. In addition, NIL-T lines accumulated less Cl^-^ in the leaf and more in the root prior to any difference in Na^+^; in the field they accumulated less pod wall Cl^-^ than the corresponding NIL-S lines. Under non-saline field conditions, no significant differences were observed for yield related traits within each pair of NIL-T and NIL-S lines, indicating there was no yield penalty for having the *GmSALT3* gene. In contrast, under saline field conditions the NIL-T lines had significantly greater plant seed weight and 100-seed weight than the corresponding NIL-S lines, meaning *GmSALT3* conferred a yield advantage to soybean plants in salinized fields. Our results indicated that *GmSALT3* mediated regulation of both Na^+^ and Cl^-^ accumulation in soybean, and contributes to improved soybean yield through maintaining a higher seed weight under saline stress.

## Introduction

Salinity is a major abiotic stress that reduces crop productivity, with the extent of agricultural land salinization increasing due to climate change and poor land management (Takeda and Matsuoka, 2008). Worldwide, more than 40% of irrigated agricultural land has been predicted to be soon affected by salinity (Munns and Gilliham, 2015). To ensure food security into the future, crops with improved tolerance to salt stress will be required. To speed up the process of creating a new generation of stress tolerant elite crop lines, stress related genes should be used in pre-breeding research. The robustness of the stress related genes can then evaluate in the field prior to the release of new varieties to farmers. Several significant gains in abiotic stress tolerance of crops have been made through manipulating their ion transport properties through such approaches (Schroeder et al., 2013). For example, wheat grain yield in saline fields was improved by up to 25% through the introduction of a root localized Na^+^ transporter via marker-assisted breeding (Munns et al., 2012).

Crop plants differ greatly in their salinity tolerance as reflected in their different growth responses at different growth stages (Foolad and Lin, 1997; Foolad, 1999; Takeda and Matsuoka, 2008). Studies on the mechanism of salt tolerance in soybean have focused mainly on seedling ion homeostasis, especially on the relative accumulation of Na^+^, Cl^-^ and K^+^ (Abel and MacKenzie, 1964; Läuchli and Wieneke, 1979; Pantalone et al., 1997; An et al., 2002). *Glycine max* seedlings under NaCl stress have been reported to have sensitivity to both Cl^-^ and Na^+^, while *Glycine soja*, a wild relative has been demonstrated to have strong Cl^-^ tolerance (Umezawa et al., 2000; Luo et al., 2005; Chen and Yu, 2007). It was reported about half century ago that the salt tolerance of soybean was controlled by a single dominant allele q*NaCl3 (Ncl)* (Abel, 1969). A major QTL or dominant locus on soybean chromosome 3 was identified and validated by several research groups in both cultivated and wild soybean (Lee et al., 2004; Chen et al., 2008; Hamwieh and Xu, 2008; Hamwieh et al., 2011; Ha et al., 2013; Guan et al., 2014a). A salt candidate gene proposed to underpin this locus was identified from the wild soybean *G. soja*, accession W05, (*Glysoja01g005509)* by using a whole-genome sequencing approach (Qi et al., 2014). This paper named the homologous gene in soybean (*Glyma03g32900.1*), *GmCHX1*, after its putative function as a cation/H^+^ exchanger, and proposed that it improved soybean salt tolerance after functional tests in tobacco BY2 cells and soybean hairy root cultures (Qi et al., 2014). Concurrent with this study, through map-based cloning from a salt tolerant Chinese soybean variety Tiefeng 8, we identified the same gene *Glyma03g32900.1*, and named it *GmSALT3* as it is likely to encode the candidate salt tolerance-associated gene on chromosome 3 (Guan et al., 2014b). Recently, Do et al. (2016) identified the equivalent allele from the salt tolerant Brazilian cultivar FT-Abyara, and named it *Ncl*, which is an abbreviation of the QTL (q*NaCl3*) identified by Abel (1969). The tolerant *GmSALT3* allele was found in *G. max* and *G. soja* germplasm that originated all over China but was most frequently associated with regions with saline soil conditions; however, the sensitive *GmSALT3* alleles (*Gmsalt3*) were much more prevalent in non-saline regions than saline regions (Guan et al., 2014b). It was proposed that the expression of the functional salt tolerance gene resulted in an energy burden on plants when salinity was not present (Qi et al., 2014), and this may explain its limited distribution in germplasm derived from non-saline areas (Guan et al., 2014b). Do et al. (2016) recently showed in the field using near-isogenic lines that there appeared to be no yield penalty in non-saline conditions for harboring *GmSALT3*, but a yield improvement under saline conditions. Such a property would make this gene (*CHX1/GmSALT3/Ncl*) an attractive prospect to breeders and farmers (Guan et al., 2014b), so further field testing in other soil types and different genetic background is required to confirm its potential (Do et al., 2016).

The salt tolerance of different soybean varieties has predo-minantly been evaluated prior to the identification of alleles associated with soybean salt tolerance (Läuchli and Wieneke, 1979; El-Samad and Shaddad, 1997; Umezawa et al., 2000; An et al., 2002; Essa, 2002). Isolation of *CHX1/GmSALT3/Ncl* has allowed the examination of its effects on Na^+^, K^+^ and Cl^-^ accumulation during salt stress and its effect on the salt tolerance of soybean seedlings has been conducted by at least three different research groups (Guan et al., 2014b; Qi et al., 2014; Do et al., 2016). Both transformation of the salt tolerant allele into the soybean variety Kariyutaka and its introgression into the salt-sensitive cultivar Jackson, significantly decreased the leaf Na^+^, K^+^ and Cl^-^ under 100 mM NaCl stress, and increased the soybean yield by 3.6–5.5 fold when irrigated the 5-week-old seedling with 1/4 concentration seawater (Do et al., 2016). However, the timecourse for the effect of *CHX1/GmSALT3/Ncl* on the accumulation of ions and its effects in different genetic backgrounds at different developmental stages is yet to be evaluated. Here, in order to identify the behavior of *GmSALT3* in differing genetic backgrounds and in different environments, we developed three sets of near isogenic lines (NILs) derived from progenies of 85–140 × Tiefeng 8 using marker-assisted selection for the target allele *GmSALT3* or *Gmsalt3*. These NILs were used to study whether *GmSALT3* had equivalent salinity tolerance in differing genetic backgrounds, to assess the effect of *GmSALT3* on salt tolerance at the emergence stage and the exclusion of Na^+^ and Cl^-^ in both seedling and mature seeds, to determine whether *GmSALT3* positively impacted soybean yield under saline field conditions and to further examine whether *GmSALT3* conferred a yield penalty to soybean yield under non-saline field conditions.

## Materials and Methods

### Development of Near Isogenic Lines by Marker-Assisted Selection

**FIGURE 1:**
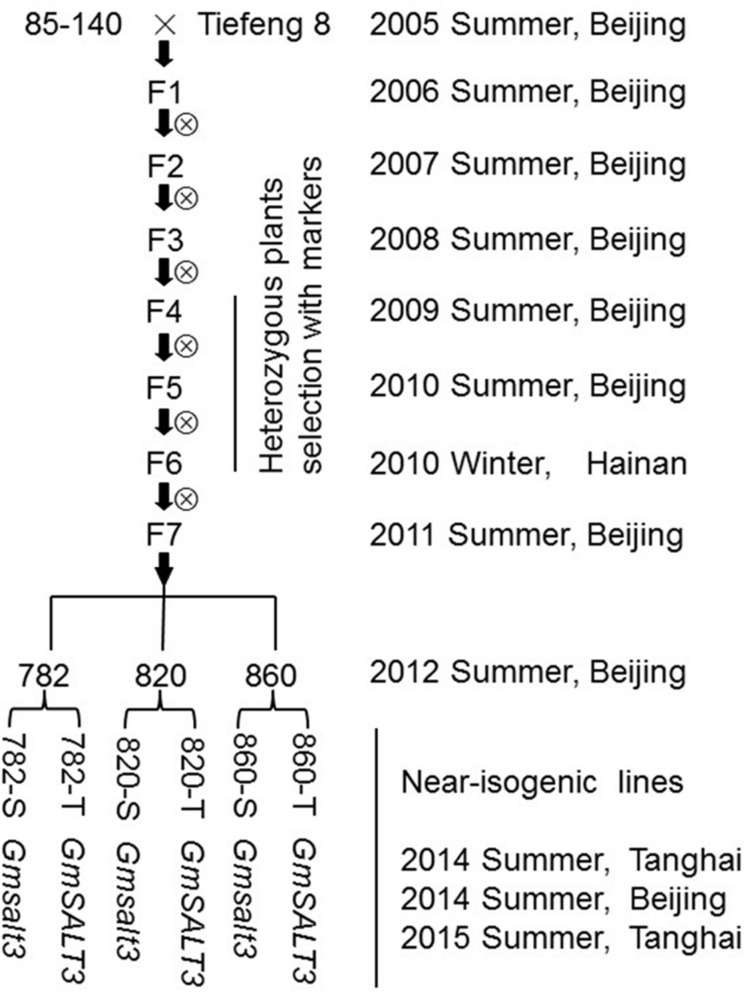
**Schematic diagram of the near isogenic lines (NILs) development by marker-assisted selection.** Target region selection was employed from generation F_4_ to F_6_. The NILs (782, 820, and 860) separated at the *GmSALT3* locus were selected by using molecular markers from the progenies of three F_6:7_ families.

A F_2:3_ population made by crossing a salt-sensitive soybean variety (85–140) and salt-tolerant variety (Tiefeng 8) was used to map the single dominant salt tolerance gene *GmSALT3* (Guan et al., 2014a). A SSR marker ssr_3_1310, tightly linked to the salt tolerance gene was used to screen heterozygous individuals at the F_4_ generation. Three heterozygous individuals, named 782, 820, and 860, were self-pollinated and screened by molecular markers over the F_5_ to F_6_ generations. A molecular marker *GmSALT3*-InDel, which was developed according to variation in the *GmSALT3* gene, was used to select three sets of different allele-containing NILs including 782-T (*GmSALT3*), 782-S (*Gmsalt3*), 820-T (*GmSALT3*), 820-S (*Gmsalt3*), 820-T (*GmSALT3*), 820-S (*Gmsalt3*), at the F_7_ generation in 2011 (**Figure 1**).

### Genetic Background Analysis of Three NIL Sets

**FIGURE 2:**
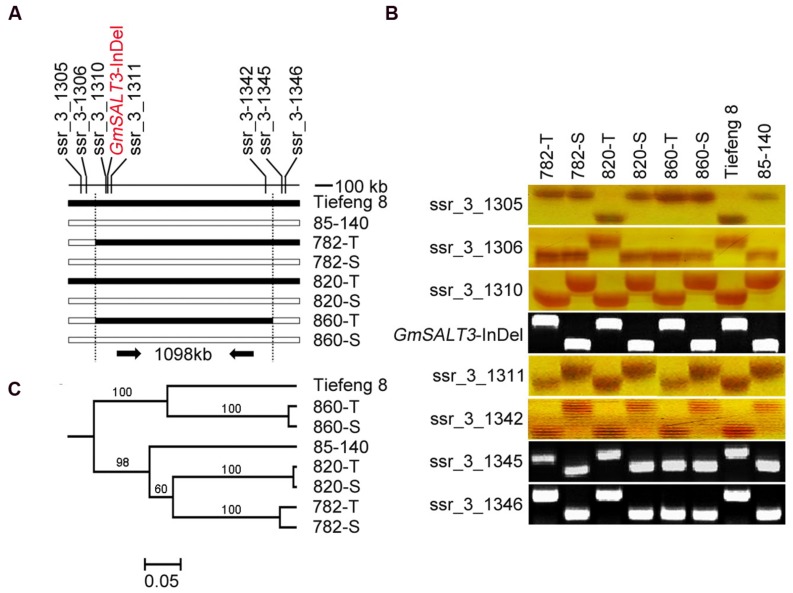
**Evaluation of a common region around *GmSALT3* and genetic backgrounds of three sets of NILs. (A)** Localization of eight markers used for common region testing of three sets of NILs, black bars indicate the same genotype with Tiefeng 8 and the white bars indicate the same genotype with 85–140. The functional marker *GmSALT3*-InDel was developed from the variation within the coding region of the target gene *GmSALT3*. **(B)** PCR assays of eight markers on chromosome 3 of NILs and their parents. **(C)** Phylogenetic relationship of NILs and their parents based on the data of 147 sets of SSR markers using the UPGMA method, the bootstrap values (%) are indicated above the node.

Eight markers covering a 1098 kb genomic region flanking the *GmSALT3/salt3* locus were used to test for the presence of the common fragment in three NIL sets (**Figure 2A**). Seven of these markers are SSR markers from SoyBase^1^. *GmSALT3*-InDel is a functional marker developed to categorize the polymorphism found between Tiefeng 8 and 85–140 in the coding region of *GmSALT3/salt3*. To evaluate the relationship between the NILs and with their original parents, 147 SSR markers that were polymorphic between Tiefeng 8 and 85–140, were selected from 342 SSR markers distributed across all 20 chromosomes (Supplementary Figure S1).

### Genotyping

For genotyping of the NILs, DNA was extracted from leaves using a Genomic DNA Purification Kit following manufacturers instructions (Thermo scientific, Lithuania). PCR was performed in a 20 μL reaction mixture containing 80 ng genomic DNA, 2 μL 10 × EasyTaq Buffer (with Mg^2+^), 1.5 μL 2.5 mmol L^-1^ dNTPs, 2 μL each of 2 μmol L^-1^ primer stock, and 1 U EasyTaq DNA Polymerase (TransGen Biotech, Beijing, China) under the following conditions: 95°C for 5 min, and then 35 cycles at 95°C for 30 s, 55°C for 30 s, and 72°C for 50 s, followed by a final extension of 5 min at 72°C. The PCR products were separated on a 1.5% agarose gel or a 6% denaturing polyacrylamide gel.

### Treatment and Phenotyping

#### NaCl Treatment and Ion Measurement

To clarify the effect of *GmSALT3* on salt tolerance at the seedling stage, 12 seeds of a single line were sown at a depth of 2.5 cm in a 6 × 6 × 8-cm pot filled with vermiculite and thinned to five seedlings per pot after 5 days; one pot was considered a replicate (i.e., the mean data from all five plants in the pot per parameter). The experimental design was completely randomized and comprised of two treatments × six genotypes with three replications, giving a total of 36 pots/replicates overall for all treatments and lines. Plants were grown in a growth chamber (RXZ-500D; Ningbo Jiangnan Instrument, China), with a day length of 16 h (with a light-emitting diode light source at 400 μmol m^-2^ s^-1^) at 28°C, and 8 h dark at 25°C, with 60% relative humidity throughout. Every treatment group (salt or control) consisted of 18 pots (9 NIL-T and 9 NIL-S) placed in a 46 × 32 × 10-cm tray, and 3.6 L of water was added to the tray. The irrigation water available in the growth facility had an electrical conductivity (EC) of 0.4 dS m^-1^ and contained potassium, calcium, sodium and magnesium in the water at a concentration of 0.06, 0.86, 0.43, and 0.46 mmol L^-1^, respectively, as determined by ICP-AES analysis (Thermo-Jarrell Ash; IRIS Advantage) and 0.46 and 0.02 mmol L^-1^ for fluoride and chloride respectively, as determined by ion chromatography (ICS-1600, Thermo Fisher Scientific). Water and treatment solutions were always added to the tray so plants accessed the solution from the bottom of the pots rather than watering the top of the pots. From the fourth day after sowing (DAS) 1.5 L of this water was added to the tray every 3 days until a salinity treatment was initiated. When the unifoliate leaves of plants were fully expanded at 12 DAS, 1.5 L of NaCl solution at an EC of 17.8 dS m^-1^ (i.e., 200 mmol L^-1^) was added to one tray, the second dose of 1.5 L of 200 mmol L^-1^ NaCl solution was applied 14 DAS; the same volume of irrigation water was applied to the tray of the control plants. Thereafter, each tray was watered every 3 days with 1.5 L of irrigation water. Twenty-two DAS, the pots were inverted and the plants and vermiculite were carefully extracted so to minimize damage to the root. Then, the roots were quickly washed with deionised water (<15 s) to remove the vermiculite and other soluble components, and blotted dry with paper towel. Root, stem, hypocotyl and unifoliate leaves of five plants in each treatment were harvested separately and placed in labeled paper bags.

To evaluate the effect of *GmSALT3* on accumulation of Na^+^ and Cl^-^, NIL-820 and NIL-860 lines which showed a high similarity of genetic background between each NIL-T and NIL-S were selected. Seeds of NILs were sown in pots as described above. There were three replicate pots of each genotype in each sample collection time point. After the unifoliate leaves of plants were fully expanded, 90 mL of 200 mmol L^-1^ NaCl solution was added 12 and 14 DAS from the bottom of each pot, respectively, after which 90 mL of irrigation water was applied at the bottom of each pot every 3 days. Plants were harvested at 0, 1, 3, 5, 7, and 10 days after beginning the NaCl treatment. The roots were washed gently with deionized water as described above and plants were dissected into roots, hypocotyl, stem and leaves.

#### Seedling Emergence

To understand whether *GmSALT3* had a positive effect on salt tolerance at the emergence stage, 10 seeds of each NIL were sown 2.5 cm deep in a 6 × 6 × 8-cm pot filled with vermiculite. Each treatment consisting of six soybean lines with three pots per line, giving a total of 18 pots, were placed in a 46 × 32 × 10-cm tray, and 3.6 L of irrigation water or NaCl solution at EC of 10.6 dS m^-1^ (i.e., 100 mmol L^-1^) or 17.8 dS m^-1^ (i.e., 200 mmol L^-1^) was applied to the tray. Four days after the first treatment, all plants were watered with 1.5 L irrigation water every 3 days. As mentioned above, the NaCl solution and water were supplied from the bottom of the pots. The experiment was conducted in the growth cabinets with conditions as described above. Emergence was counted 10 DAS. Relative emergence rate was the ratio of emerged seedlings under salt stress compared to the corresponding emerged seedlings under control (non-saline) condition. Fifteen DAS, the pots were inverted and the plants and vermiculite were carefully extracted to minimize damage to the roots. Then, the roots were quickly washed with deionized water to remove vermiculite and other soluble components, and blotted dry with paper towel. Fresh mass and shoot length of the seedlings were obtained through their measurement on weighing scales and manually using a ruler.

### Yield of NILs under Saline and Non-saline Field Conditions

#### Beijing 2014

For the comparison of agronomic traits for each pair of NILs under non-saline conditions the three sets of NILs were planted in a non-saline field in Shunyi Experimental Station, Shunyi, Beijing, China (latitude 40°13′ N, longitude 116°65′ E). Soybean seeds were sown at a depth of 3 cm on 19 June 2014. The rainfall during the June to October at Shunyi in 2014 was 394.0 mm (data from China Meteorological Data Sharing Service System). The soil was fertilized with 375 kg ha^-1^ (NH_4_)_2_HPO_4_ and 150 kg ha^-1^ KCl before sowing. Soybean seeds were planted in 1.5 m long three-row plots with three replications, with row spacing of 50 cm and a spacing of 8 cm between plants. The soybean plants were irrigated once in August during seed filling. At maturity, the plants were cut from the surface of the soil. Fifteen plants of each line were bulked per replication three times for agronomic trait evaluation.

#### Hebei 2014, 2015

The saline soil trials were conducted in Tanghai county (Hebei Province, China), along the Bohai coast, where 40.3% of the fields had saline soil with an average soluble salt of more than 2 g kg^-1^ (Zhang et al., 2012). The saline field used in this experiment was located in No. 11 farm of Tanghai county (latitude 39°27′ N, longitude 118°45′ E). White crusts of salt were observed on the soil surface. The soluble salt concentration was evaluated by measuring the EC of the aqueous extract of the soil cores down to 25–30 cm before sowing the soybean. After coring, the soil was air-dried and passed through a 2 mm mesh sieve. The EC of the 1: 5 soil: water (w/v) mixture (in terms of g water per g dry soil) was measured by using a digital conductivity meter DDS-11A (Leici Instrument Inc., Shanghai, China). The pH was determined with a pH electrode PHS-3C at a soil : water ratio of 1: 5 (w/v) after 30 min in suspension. For the 2014 field trial, the EC and pH of soil : water (1:5) was 0.6 dS m^-1^ and 8.18, the soybean NILs for the field experiment were planted on the 10th June 2014 and harvested on the 20th October, with approximately 15 seeds per row (1.0 m long). The 2015 field trail was located in the same paddock and about 25 m south of the 2014 trial, the EC and pH of soil: water (1: 5) was 0.7 dS m^-1^ and 7.6, the soybean NILs for the field experiment were planted on the 25th June 2015 and harvested the 14th October, with approximately 25 seeds per row (1.5 m long). A completely random design with three replications was used, with each plot containing three rows of each line and no fertilizer was applied. The rainfall during June and October at Tanghai was 265.4 and 396.0 mm in 2014 and 2015, respectively (data from China Meteorological Data Sharing Service System), and the soybean plants were grown without supplemental irrigation. At maturity, the plants were cut from the surface of the soil. Fifteen plants of each line were bulked per replication three times for agronomic trait evaluation.

### Tissue Ion Analysis

All plant samples were dried at 75°C for 3 days in a forced air oven. Seeds and pod walls from 2015 saline field trial were oven-dried for 3 days at 40°C. Samples were ground to a fine powder using metal beads in a SPEX 2000 Geno/Grinder (SPEX CertiPrep, USA) and 0.1 g of subsample was extracted with 10 mL of 100 mmol L^-1^ acetic acid at 90°C for 3 h in a water bath shaker. Sodium and potassium concentration was measured with an atomic absorption spectrophotometer SOLAAR s2 (Thermo Elemental, Waltham, MA, USA), to give the concentrations of the two ions in different tissues. Chloride was measured with Chloride Analyzer (Model 926, Sherwood, UK).

### Data Analysis

One-way ANOVA followed by Tukey’s HSD *post hoc* test was performed on the analysis of ions concentration and yield related trait comparison using Prism 3.0 software (GraphPad Software, La Jolla, CA, USA), lowercase letters were used to indicate statistically significance differences between groups at *P* < 0.05. Student’s *t*-tests were used for other data analysis, single or double asterisks indicated statistical significance corresponding to *P* < 0.05 or *P* < 0.01, respectively. The neighbor-joining tree was constructed by using Power Marker 3.23 with 1000 bootstrap replicates (Liu and Muse, 2005)^2^, and MEGA (Tamura et al., 2007) was used to view the dendrogram tree.

## Results

### Genomic Composition of Three Sets of NILs

To evaluate the impact of *GmSALT3* on soybean growth in differing genetic backgrounds, three sets of NILs were developed from the F_7_ progeny of a cross (85–140 × Tiefeng 8) after three generations of marker-assisted self-pollination (**Figure 1**). The common interval, 1098 kb on chromosome 3, of the three NIL-T lines was determined using eight markers including the functional marker in *GmSALT3* (**Figures 2A,B**). The lines (782-T, 820-T, 820-T) carrying the functional Tiefeng 8 type allele *GmSALT3*, were named NIL-T and the other corresponding lines (782-S, 820-S, 820-S) carrying the non-functional allele *Gmsalt3* from 85–140 were named NIL-S.

To estimate the similarity of their wider genetic backgrounds, 147 genome-wide polymorphic SSR markers were used to genotype the three sets of NILs (Supplementary Figure S1). The SSR assay showed that the genetic similarity between 860-S and 860-T was 98.0%, between 782-T and 782-S was 95.6%, and between 820-T and 820-S was 99.3%. Phylogenic analysis showed NIL-860 shared a similarity of 64% with Tiefeng 8; the other two sets of NIL-782 and NIL-820 shared similarity of 65 and 55% with 85–140, respectively (**Figure 2C**).

### Na^+^ Accumulation in Different NILs Was Affected by Genetic Background

**FIGURE 3:**
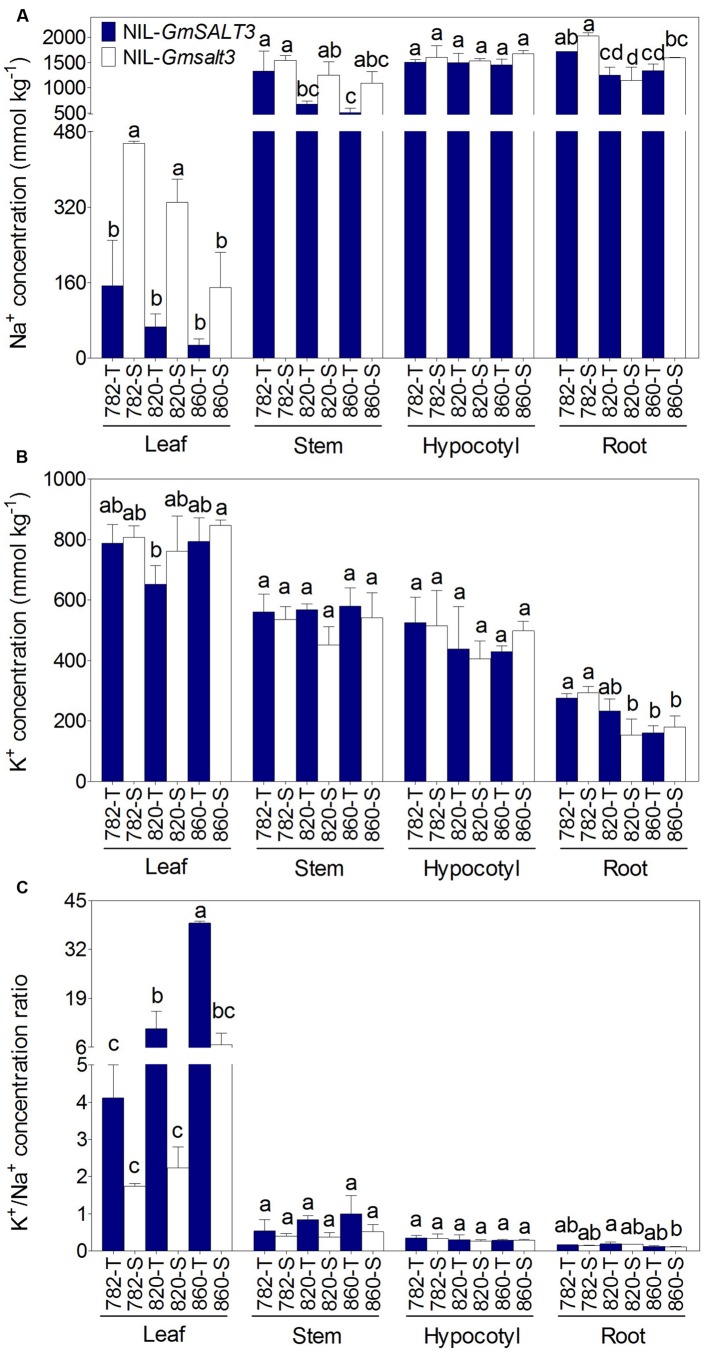
**Ion concentration (dry mass) in three sets of NILs after 10 days of NaCl stress (200 mmol L^-1^; EC = 17.8 dS m^-1^). (A)** Tissue concentration of Na^+^ in leaf, stem, hypocotyl and root of three sets of NILs. **(B)** Concentration of K^+^ in leaf, stem, hypocotyl and root of three sets of NILs. **(C)** The K^+^/Na^+^ ratio in leaf, stem, hypocotyl and root of three sets of NILs. Data are means of three replicates consisting of the mean values for five plants grown in the same pot ± SD (*n* = 3). Different letters indicate statistically significant differences between NIL lines for each tissue (one-way ANOVA followed by Tukey’s HSD *post hoc* test, *P* < 0.05).

Na^+^ and K^+^ accumulation was explored in the three sets of NILs after NaCl stress for 10 days. Significantly lower Na^+^ concentration was observed in both leaf and stem of NIL-T lines compared with their NIL-S plants, regardless of their genetic backgrounds (**Figure 3A**). Given that the different NIL-T had the same *GmSALT3* allele, but differing genetic backgrounds, we compared the effect of the genetic backgrounds on Na^+^ accumulation. We found that 782-T accumulated relatively more Na^+^ than 820-T and 860-T in both the stem and leaf samples, indicating genetic loci other than *GmSALT3* might influence Na^+^ accumulation. This is also supported by the fact that the corresponding NIL-S (820-S and 860-S) also accumulated less Na^+^ in the stem, leaf and root than that of 782-S (**Figure 3A**). No significant differences in leaf K^+^ concentration was observed within each pair of NILs (**Figure 3B**). This indicated that the *GmSALT3* had little effect on the regulation of K^+^ homeostasis and that the regulation of K^+^/Na^+^ balance in the shoots was mostly dependent on the accumulation of Na^+^ in shoots (**Figure 3C**).

### A Detailed Analysis of NIL-820 and NIL-860 Reveals *GmSALT3* Also Affects Cl^-^ Accumulation

**FIGURE 4:**
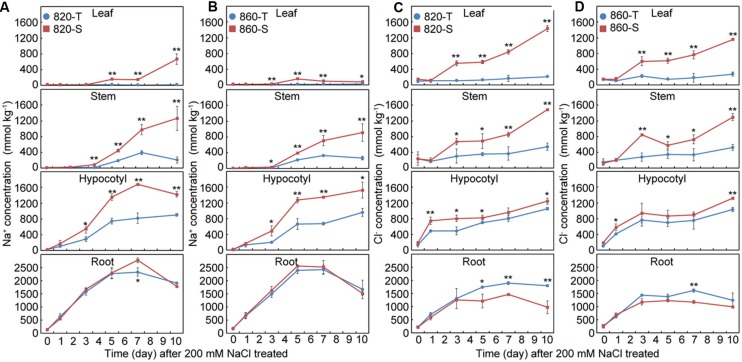
**Time-course of ion concentration (dry mass) in different tissues of NIL-820 and NIL-860 during 10 days of NaCl stress (200 mmol L^-1^; EC = 17.8 dS m^-1^).** Na^+^ concentration in leaf, stem, hypocotyl and root of NIL-820 **(A)**, and NIL-860 **(B)**, or Cl^-^ concentration in leaves, stems, hypocotyls and roots of NIL-820 **(C)**, and NIL-860 **(D)**. Data are means of three replicates consisting of the mean values for five plants grown in the same pot ± SD. Student’s *t*-test was used to compare NIL-T and NIL-S at the same time point (^∗^*P* < 0.05, ^∗∗^*P* < 0.01).

As the effect of *GmSALT3* was similar on shoot Na^+^ accumulation between each set of NILs, we undertook a closer examination of Na^+^ and Cl^-^ accumulation in NIL-820 and NIL-860 following a NaCl treatment over 10 days (**Figure 4**). Over the first 3 days of NaCl treatment, Na^+^ and Cl^-^ accumulation in the roots was equal for both tolerant and sensitive genotypes of NIL-820 and NIL-860 (**Figure 4**), whereas the Cl^-^ concentration in roots of 820-S and 860-S was significantly lower than in the 820-T and 860-T lines after 5 and 7 days respectively (**Figures 4C,D**). In both NIL-T and NIL-S roots the Na^+^ and Cl^-^ concentration plateaued after 5 – 7 days of NaCl treatment, and the accumulation of Na^+^ was higher than that of Cl^-^ (**Figure 4**). In the aerial tissues of NIL-T soybean, Na^+^ accumulation occurred later than that of Cl^-^. Accumulation of Cl^-^ was consistently significantly greater in the hypocotyls of both NIL-S compared to their respective NIL-T after 1 day of salt treatment, whereas this was only the case for Na^+^ in the first instance after 3 days (**Figure 4**). The soybean leaves accumulated more Cl^-^ than that of Na^+^. The concentration of Na^+^ in NIL lines decreased from the root to leaf, but this was not the case for Cl^-^ concentration in NIL-S lines, which was high across all shoot organs (**Figures 4C,D**). These results indicate that *GmSALT3* influences both Na^+^ and Cl^-^ accumulation in soybean plants under a salt treatment.

### *GmSALT3* Has Little Effect on Soybean Growth at the Emergence Stage under Saline Stress

**FIGURE 5:**
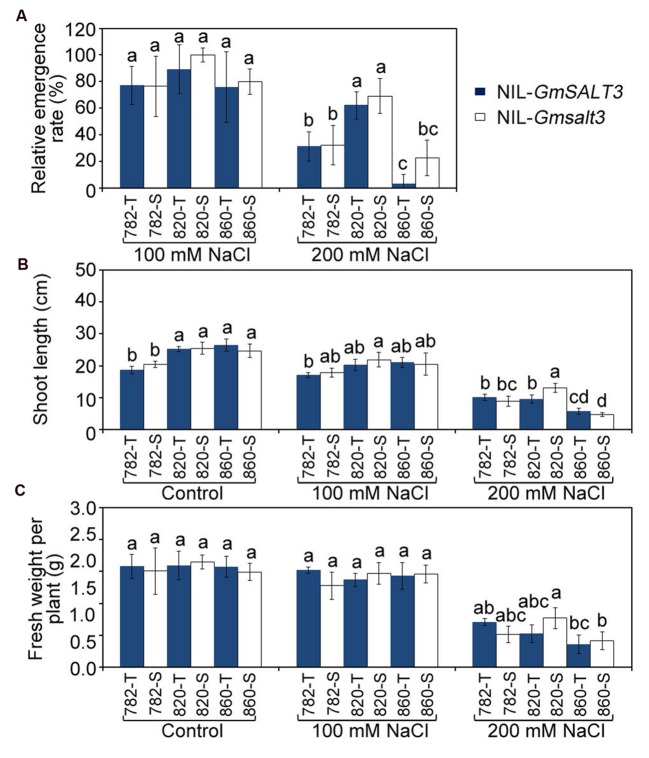
**Emergence rate after 10 days and early vigor after 15 days of three sets of NILs under control and NaCl stress conditions. (A)** The relative emergence rate of three sets of NILs under NaCl stresses (EC = 10.6 dS m^-1^, 17.8 dS m^-1^). **(B)** The seedling length of NIL lines under control and NaCl stress (EC = 10.6 dS m^-1^, 17.8 dS m^-1^). **(C)** Average plant weight of three sets of NILs under control and NaCl stress (EC = 10.6 dS m^-1^, 17.8 dS m^-1^). Data are means of three replicates ± SD (*n* = 3). Different letters indicate statistically significant differences between NIL lines (one-way ANOVA followed by Tukey’s HSD *post hoc* test, *P* < 0.05).

We analyzed the relative emergence rate and early vigor of the three NIL sets to see if *GmSALT3* also affected the salt tolerance of soybean at the emergence stage (**Figure 5**, Supplementary Figure S2). We quantified the effect of *GmSALT3* gene on emergence by using relative emergence rate after 10 days. The relative emergence rates were greater for all genotypes when treated with 100 mmol L^-1^ NaCl (EC of 10.6 dS m^-1^); compared to the NaCl solution of 200 mmol L^-1^ NaCl (EC of 17.8 dS m^-1^). Whilst NIL-820 showed a higher relative emergence rate than the other two sets of NILs when treated with 200 mmol L^-1^ NaCl (**Figure 5A**), no significant differences in relative emergence rate within each set of NILs was observed when they were treated with 100 and 200 mmol L^-1^ NaCl solution (**Figure 5A**). Early vigor was estimated by measuring shoot length and fresh weight 15 DAS. Early seedling vigor showed no difference within each set of NILs under saline and non-saline conditions except for the 820 NILs, where 820-S had greater shoot length than 820-T treated with 200 mmol L^-1^ NaCl (**Figures 5B,C**).

### *GmSALT3* Has a Positive Effect on the Yield of NIL-T under Saline Stress

**FIGURE 6:**
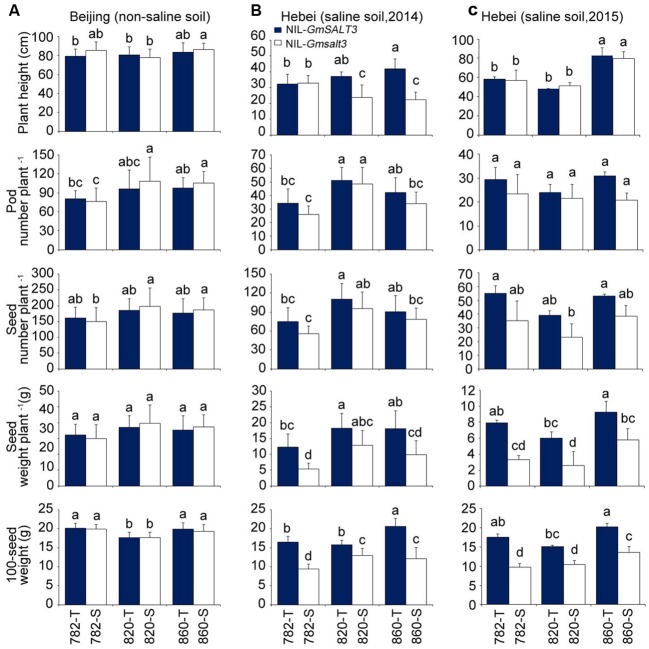
**Yield related traits comparison for three sets of NILs grown in saline and non-saline soil conditions. (A)** Plant height, pod number per plant, seed number per plant, seed weight per plant and 100-seed weight of three sets of NILs grown in non-saline soil (Shunyi, Beijing). Data are means of three replicates consisting of mean data from 15 plants per replicate ± SD (*n* = 3). **(B)** Plant height, pod number per plant, seed number per plant, seed weight per plant and 100-seed weight of three sets of NILs grown in saline soil (Tanghai, Hebei province, 2014). **(C)** Plant height, pod number per plant, seed number per plant, seed weight per plant and 100-seed weight of three sets of NILs grown in saline soil (Tanghai, Hebei province, 2015). Data are means of three replicates consisting of mean data from 15 plants per replicate ± SD (*n* = 3). Different letters indicate statistically significant differences between NIL lines (one-way ANOVA followed by Tukey’s HSD *post hoc* test, *P* < 0.05).

When the three sets of NILs were grown in non-saline field conditions (Shunyi, Beijing), NIL-T lines containing *GmSALT3* had a similar yield to the corresponding NIL-S lines containing *Gmsalt3* for the yield parameters of pod number, seed number, seed weight and 100-seed weight (**Figure 6A**). The yield related components of both NIL-T lines and NIL-S lines were substantially affected by salinity stress when NILs were grown on saline soil (Tanghai, Hebei province) with the respective NIL-S for each set of lines being noticeable smaller at harvest maturity (Supplementary Figure S3). In terms of the plant height, 820-T and 860-T were significantly taller than that of 820-S and 860-S when grown on saline soil whereas 782-T and 782-S were similar in size at Tanghai in 2014; while in 2015 saline field trial plant height was equal between NIL pairs (**Figure 6C**). Under saline conditions no significant difference in pod number and seed number were observed between each pair of NIL-T and NIL-S. However, the NIL-T lines containing *GmSALT3* had significantly greater seed weight and 100-seed weight than related NIL-S lines regardless genetic backgrounds under saline conditions (**Figures 6B,C**). The seed weight of NIL-S lines was 30–57% lower than corresponding NIL-T lines in 2014 saline field trial, and 37–58% lower in 2015 saline field trail. The mean above ground dry mass of the NIL-S lines was lower than the corresponding NIL-T lines, but a significant difference was only observed between 782-T and 782-S (Supplementary Figure S4).

### Effects of *GmSALT3* on Na^+^, K^+^ and Cl^-^ Concentration in Seeds and Pod Walls under Saline Stress

**FIGURE 7:**
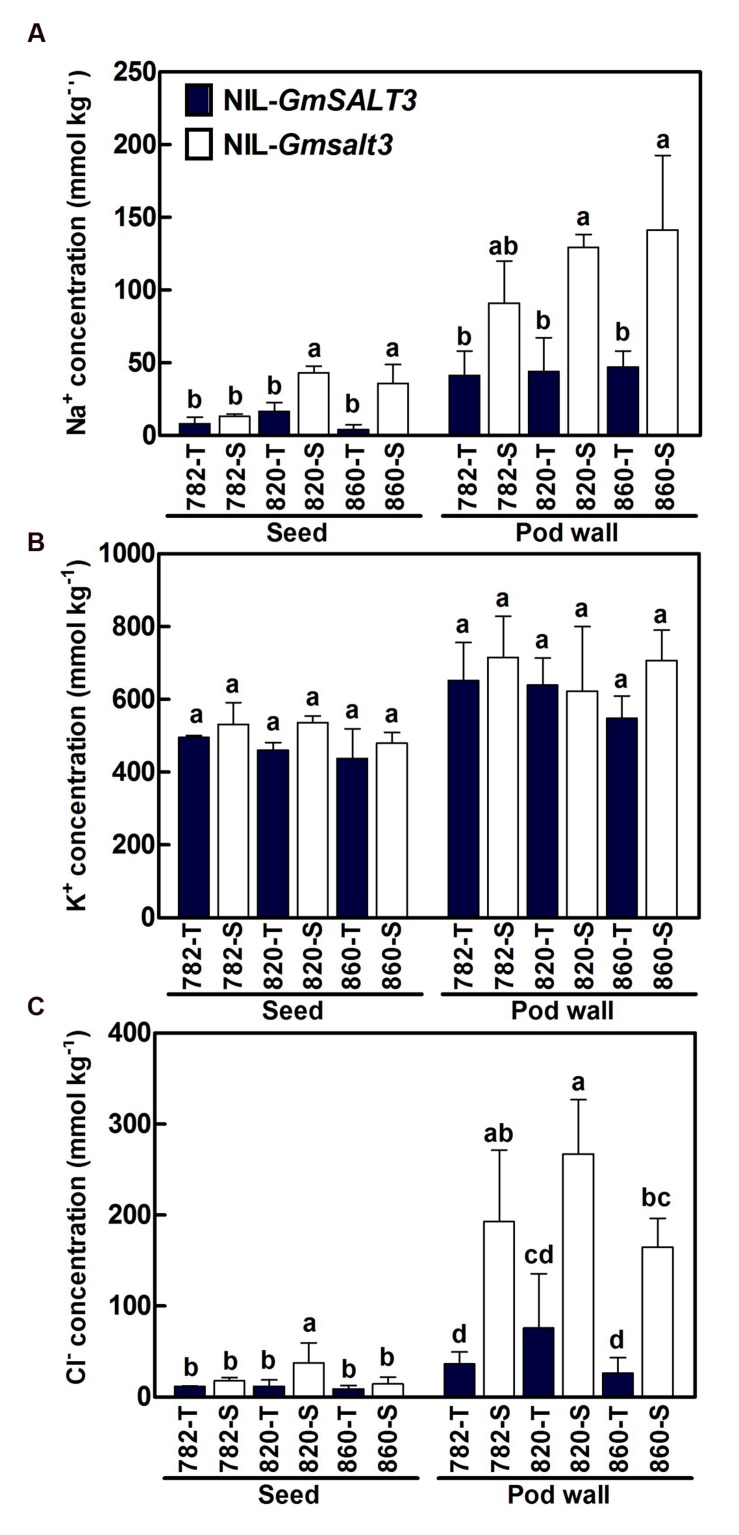
**Mean Na^+^ (A), K^+^ (B), Cl^-^ (C) concentrations (dry mass) in seeds and pod walls of three sets of NILs grown on saline field (2015, Tanghai).** Data are means of three replicates consisting of the mean values for 15 plants for per replicate ± SD (*n* = 3). Different letters indicate statistically significant differences between NIL lines for each tissue (one-way ANOVA followed by Tukey’s HSD *post hoc* test, *P* < 0.05).

In the saline field (Tanghai, 2015), the Na^+^ concentration in seeds and pod walls of three sets of NILs ranged from 4.1 to 42.9 mmol kg^-1^ and 41.4 to 141.4 mmol kg^-1^ dry mass, respectively. Na^+^ concentrations in seeds and pod walls of three NIL-T lines were significantly lower than the corresponding NIL-S lines, except for the Na^+^ concentrations between 782-T and 782-S (**Figure 7A**). Pod walls contained higher K^+^ concentrations than seeds, but no significant differences were observed for K^+^ concentrations in both seeds and pod walls among genotypes grown in saline soil (**Figure 7B**). All three NIL-T lines grown on saline field had low seed Cl^-^ concentrations ranging from 8.8 to 11.8 mmol kg^-1^ dry mass. The seed Cl^-^ concentrations in NIL-S lines were greater by 1.5, 3.2, and 1.6 times in 782-S, 820-S, and 860-S compared to their corresponding NIL-T lines, respectively (**Figure 7**), but a significant difference was only observed between 820-T and 820-S. The Cl^-^ concentrations in pod wall of 782-T, 820-T and 860-T were 48.8, 101.2, and 35.1 mmol kg^-1^ dry mass, respectively. Compared to NIL-T lines, the Cl^-^ concentrations in pod wall of 782-S, 820-S, and 860-S were greater by 5.3, 3.5, and 6.3 times. As a result the NIL-T lines carrying the functional *GmSALT3* allele accumulated less Cl^-^ in seeds and pod walls than their corresponding NIL-S lines under saline stress.

## Discussion

Previous studies have mostly evaluated the salt tolerance of soybean at the seedling stage. Such studies have repeatedly identified the same major QTL independently (Lee et al., 2004, 2009; Chen et al., 2008; Hamwieh and Xu, 2008; Hamwieh et al., 2011; Ha et al., 2013; Guan et al., 2014a), with only a few studies focusing on QTL mapping at the germination stage (Zhang et al., 2014). Recently, we and other groups have isolated a dominant salt tolerance gene *CHX1*/*GmSALT3/Ncl* located on chromosome 3 (Guan et al., 2014b; Qi et al., 2014; Do et al., 2016). Here, we successfully created three sets of NILs containing *GmSALT3* and *Gmsalt3*, respectively. The three tolerant NILs had differing genetic backgrounds whilst containing a common ~1000 kb region including the *GmSALT3* gene in NIL-T lines or *Gmsalt3* in NIL-S lines. The NILs differed at the major salt tolerant locus are suitable for assessing the impact of tolerant allele on ions accumulation and yield related traits.

Negative correlations between leaf Na^+^ concentration and salt tolerance have been widely observed in soybean and other crop species (Schachtman and Munns, 1992; Kao et al., 2006). Here, significantly higher Na^+^ concentrations and a lower K^+^/Na^+^ ratio were observed in leaves of NIL-S lines compared with the corresponding NIL-T lines when salt treated (**Figure 3A**). Whereas, there was no observable difference in leaf Na^+^ concentration and K^+^/Na^+^ ratio between each pair of NIL-T and NIL-S lines under non-saline conditions (Supplementary Figure S5). This again strengthens the evidence that *GmSALT3* exerts a positive effect on soybean salt tolerance. Furthermore, the significantly lower Na^+^ concentration observed in the leaf of 860-S compared to that of 820-S indicated that the degree of Na^+^ accumulation exerted by genetic elements in addition to *GmSALT3* deserve further investigation in this set of NILs (**Figures 3A** and **4A,B**). It is suggested that Na^+^ inhibits enzyme activity through competing with K^+^ for binding sites at high concentration (Tester and Davenport, 2003; Munns and Tester, 2008). The exclusion of Na^+^ in plant shoots is controlled by either xylem loading or phloem re-translocation to prevent the toxic accumulation of Na^+^ in photosynthetic tissues (Maathuis et al., 2014). Since the export of Na^+^ through phloem in soybean is not sufficient to control the accumulation of Na^+^ in leaves, the salt tolerance of soybean may depend on its ability of Na^+^ delivery into xylem (Durand and Lacan, 1994). The K^+^ concentration in each tissue of each NIL was not consistently related to changes in Na^+^, which has been also observed in wheat and barley (Genc et al., 2007; Tavakkoli et al., 2011).

Cl^-^ plays a major role in membrane potential charge balance and in pH control (Teakle and Tyerman, 2010). A limited number of studies have shown that Cl^-^ concentration was correlated with salt sensitivity in soybean (Abel and MacKenzie, 1964; Läuchli and Wieneke, 1979; Valencia et al., 2008). Luo et al. (2005) documented that in cultivated soybean seedlings, Cl^-^ was more toxic than Na^+^. Although the same osmotic potential (-0.68 MPa) was used for treatment of Na^+^, Cl^-^ and NaCl in the experiments, the altered concentration of other ions (K^+^, Ca^2+^ and Mg^2+^) in the solution may affect the plant response (Luo et al., 2005). In faba bean, Na^+^ and Cl^-^ limits the plant growth simultaneously but through different mechanisms (Tavakkoli et al., 2010). It has been reported that Na^+^ and Cl^-^ had additive effects on the growth of barley (Tavakkoli et al., 2011). Plants containing *GmSALT3* had much less Cl^-^ accumulating in leaves and more in the roots when salt treated. The significantly lower Na^+^ and Cl^-^ in both 820-T and 860-T compared with 820-S and 860-S strongly suggests that *GmSALT3* has a strong effect on both Na^+^ and Cl^-^ exclusion (**Figures 4A,B**). Interestingly the concentration difference of Cl^-^ in aerial tissues between NIL-T and NIL-S appeared prior to that of Na^+^. Furthermore, it suggests that innate Na^+^ exclusion in the NIL-S is greater than that of Cl^-^ exclusion, but Na^+^ exclusion breaks down after several days of salt treatment. The difference of Na^+^ and Cl^-^ accumulations between NIL-T and NIL-S suggests the participation of *GmSALT3* in both Na^+^ and Cl^-^ homeostasis, and that Cl^-^ accumulation is decoupled from accumulation of Na^+^ (and K^+^) and occurs via a distinct mechanism. Do et al. (2016) suggested that CHX1/GmSALT3/Ncl controls Na^+^, K^+^ and Cl^-^ accumulation simultaneously, and may function as a cation-chloride cotransporter (CCC). Both the CCC in rice and *Arabidopsis* affects the transport of these three ions to the shoot; plants that lack *CCC* expression are salt sensitive (Colmenero-Flores et al., 2007; Kong et al., 2011; Henderson et al., 2015; Chen et al., 2016). Some data has been used to propose a plasma membrane localisation for rice CCC (Kong et al., 2011; Chen et al., 2016) while AtCCC and VviCCC, have both been localized to the Golgi and Trans-Golgi network, and so are unlikely to directly affect Na^+^, K^+^, and Cl^-^ transport between the root symplast and xylem apoplast and their accumulation in the shoot (Colmenero-Flores et al., 2007; Henderson et al., 2015). GmSALT3 is also predicted to be an endomembrane protein having been localized to the endoplasmic reticulum (ER) (Guan et al., 2014b); it shares this localisation with several other CHX protein family members from *Arabidopsis* (Sze et al., 2004; Padmanaban et al., 2007; Chanroj et al., 2012). The CHX proteins in *Arabidopsis* mainly function in osmotic adjustment, and K^+^ or Na^+^ homeostasis (Sze et al., 2004; Hall et al., 2006; Maresova and Sychrova, 2006; Padmanaban et al., 2007; Lu et al., 2011). Therefore, quite how GmSALT3 confers these traits of Na^+^ and Cl^-^ exclusion is still unknown and is the subject of further research.

The relationship between increased salt accumulations in leaves with decreased soybean yield has been reported (Abel and MacKenzie, 1964; Bustingorri and Lavado, 2013). However, little is known about the concentration of ions in the reproductive structures of soybean. A negative relationship between Cl^-^ accumulation in seeds with seed yield and weight has been observed in soybeans when KCl was used as fertilizer in a field experiment, indicating the soybeans suffered from chloride toxicity which appeared to come from the KCl fertilizer (Parker et al., 1983). Analysis of the ion concentration in mature seeds and pod walls of NILs grown in a saline field revealed a lower seed Na^+^ concentration in NIL-T lines compared with the corresponding NIL-S lines, but the difference was not significant between that of 782-T and 782-S; in pod walls, Na^+^ concentrations were 2.6–11.4 fold higher than in the seeds (**Figure 7A**). The difference in seed Cl^-^ concentrations between each pair of NIL-T and NIL-S were not significant, except for 820-T and 820-S, while all NIL-T lines accumulated significantly lower Cl^-^ in pod walls than that of NIL-S lines. Whether accumulation of Na^+^ and Cl^-^ in the seed and pod wall interferes directly with seed development or whether the effects are due to accumulation of salt in other parts of the plant and reducing the energy devoted to developing seeds is yet to be tested. In chickpea, a significantly higher concentration of seed sodium and potassium was observed in salt sensitive genotypes compared to tolerant ones (Turner et al., 2013). While a recent study of chickpea genotypes subjected to NaCl stress found that the changes of Na^+^ and Cl^-^ in mature seeds of salt tolerant and sensitive genotypes were not associated with salinity tolerance (Kotula et al., 2015).

The extent to which tolerance factors identified in hydroponic or pot assays under controlled conditions hold up to scrutiny in the field, where the imposition of stress and other environmental factors are more dynamic, is a common problem when translating lab research to the field (Genc et al., 2007). As such, it is important to assess plant tolerance to stress at different growth stages. We have previously identified *GmSALT3* as a dominant gene conferring salt tolerance at the seedling stage. Here, the development of NILs made it possible for us to also evaluate the function of *GmSALT3* at an emergence stage under salt stress. To mimic the natural salinity conditions occurring at our field sites, as salt is likely to be present when the seeds are sown in the field, we sowed the soybean seeds in vermiculite and watered with NaCl solution. Under NaCl stress at EC of 10.6 dS m^-1^ (i.e., 100 mmol L^-1^ NaCl) most of the NILs lines germinated at a relatively high levels, while a higher concentration of NaCl inhibited the emergence of the soybean and limited the plant growth. Under NaCl stress at EC of 17.8 dS m^-1^ (200 mmol L^-1^ NaCl) most of the cotyledons were yellow and unable to maintain turgor (Supplementary Figure S2). Previously, reduction of seed water absorption was observed when the osmotic pressure in germinating solution was increased by NaCl (Rudolfs, 1921). The reduction of emergence rate under higher salinity levels is likely to be a result of an osmotic stress (Bernstein and Hayward, 1958). No significant differences were observed within each set of NIL lines, indicating that the existence of *GmSALT3* did not increase soybean salt tolerance at the emergence stage. This is consistent with previous studies that the salt tolerance at one stage is not always correlated with that of the other growth stages (Abel and MacKenzie, 1964). Furthermore, the sensitivity of soybean seedlings to salinity has previously been proposed to be greater than that of germinating seeds, as is the case for many plant species (Rogers et al., 1995; Hosseini et al., 2002) – our research findings here seem to corroborate this statement. During QTL mapping of salt tolerance of seed germination and vegetative stages of tomato, few common QTL were observed that had significant effects at both stages (Foolad, 1999). With this in mind, when using GmSALT3 in the field it would be wise to use appropriate soil and water management practices to enhance the germination rate (Devkota et al., 2015), or to pyramid the *GmSALT3* gene with the recently identified salt tolerance related candidate genes at the germination stage (Kan et al., 2015). In our study, both NIL-820 lines showed better salt tolerance at the emergence stage compared to the other two sets of NIL lines, so these could potentially be used as a resource for improving salt-tolerance at the emergence stage (**Figure 5A**, Supplementary Figure S2).

The characterization of the different alleles of *GmSALT3* in our previous report suggested that the tolerant allele had been under significant selection pressure; it was frequently lost in non-saline environments (Guan et al., 2014b). Thus, this raises an important question of whether *GmSALT3* incurs a penalty to yield under non-saline conditions. Since most salinity affected soils are not uniformly saline or apply a constant level of salinity during the entire crop growth cycle, genes with no penalty are more appropriate for breeding salt tolerant crops (Munns et al., 2012). In our case, we found that under non-saline field conditions NIL-T lines had similar yield related traits compared to their related NIL-S lines (**Figure 6A**), indicating no yield penalty associated with the presence of *GmSALT3* allele. We therefore find no reason why *GmSALT3* may have been selected against on the basis of yield under modern farming practices. This result corroborates the recent findings of Do et al. (2016) who also found, using different soybean genetic backgrounds, that the functional *GmSALT3* allele does not harbor a yield penalty under non-saline conditions, whilst conferring improved yields under saline conditions.

The effect of GmSALT3 on soybean growth and yield was evaluated by comparing three sets of NIL lines over 2 years in Tanghai, Hebei province. Pod number and seed number per plant decreased for both NIL-T and NIL-S lines, compare with control plants, while the difference between each pair of NIL-T and NIL-S was not significant. The lower yield reduction of a super-nodulating en-b0-1 compared with its normal-nodulating parent Enrei under salinity stress was primarily due to the larger seeds number of en-b0-1 (Yasuta and Kokubun, 2014). Analysis of chloride toxicity of soybeans grown in Flatwoods soils fertilized with KCl identified an average of 25% less 100-seed weight in susceptible cultivars than that for tolerant ones (Parker et al., 1983). Significant differences in 100-seed weight were observed between each pair of NIL-T and NIL-S, suggesting that NIL-S is compromised in its ability to produce larger seeds and this contributes to the 30–58% loss of seed yield for NIL-S lines (**Figure 6**). Do et al. (2016) also showed that the *Glyma03g32900* gene cold increase soybean yield by 3.6–5.5 fold when treated with diluted seawater in Japan (Do et al., 2016), indicating the wide potential for using this gene to improve the salt tolerance of soybean.

## Conclusion

In this study, three sets of NILs differing at *GmSALT3* locus were developed through marker-assisted selection, and used to evaluate the possible effect of *GmSALT3* on ion accumulation and yield production. Our results clearly indicate that *GmSALT3* alters both Na^+^ and Cl^-^ accumulation in shoots and mature pod walls of NIL-T lines. The salt tolerance gene *GmSALT3* was found to have no penalty on soybean yield under non-saline condition and contributes to improving soybean yields through increasing seed weight in different genetic backgrounds under salinity stress in the field (**Figures 6B,C**). The salinity tolerance of NIL-T lines was related with the maintenance of seed size under salt stress, with this ability associated, at least partially, with the ability to regulate Na^+^ and Cl^-^ in both vegetative and reproductive tissues. Interestingly we found that GmSALT3 first limits Cl^-^ accumulation in the leaf and then Na^+^, through a yet to be identified mechanism. This study provides useful molecular markers for introducing the *GmSALT3* gene into breeder’s varieties; the NIL-T lines used in this experiment have the potential to be used as gene sources to accelerate breeding for improvement of salt tolerance in commercially grown soybean varieties.

## Author Contributions

LQ, RG, and MG designed experiments. YL performed development of NILs and K^+^, Na^+^ concentrations analysis. LY, JC, HH, ZL, and RC participated field experiments and data collection. YQ and XL performed the Cl^-^ concentration test. YL, LQ, RG, YQ, and MG wrote the paper. All authors read and approved the final manuscript.

## Conflict of Interest Statement

The authors declare that the research was conducted in the absence of any commercial or financial relationships that could be construed as a potential conflict of interest.

## Footnotes

1 www.soybase.org

2 http://statgen.ncsu.edu/powermarker/
