# Supplementary material for: GmSALT3, Which Confers Improved Soybean Salt Tolerance in the Field, Increases Leaf Cl- Exclusion Prior to Na+ Exclusion But Does Not Improve Early Vigor under Salinity
Source: Front Plant Sci. 2016 Sep 30;7:1485. doi: 10.3389/fpls.2016.01485 (PMC5043451; doi:10.3389/fpls.2016.01485)
Supplement: Supplementary file 1 [file Data_Sheet_1.DOCX]

**
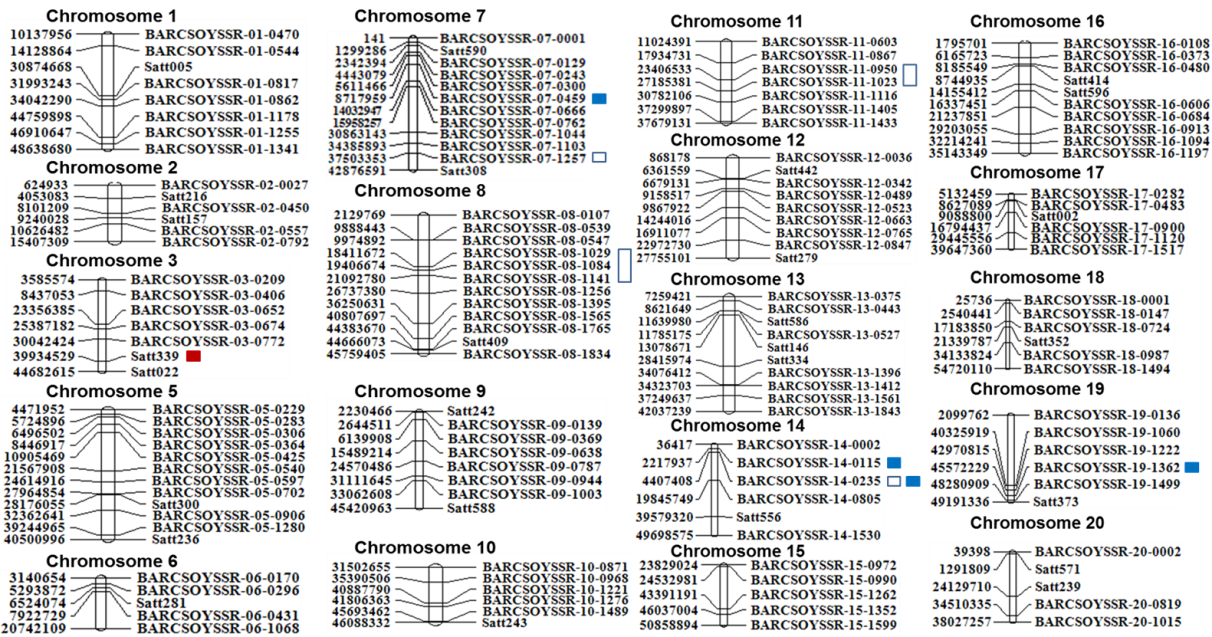
**

**Supplementary FIGURE 1.** Distribution of 147 SSR markers which are polymorphic between Tiefeng 8 and 85-140 on 19 chromosomes of soybean. The empty bars, red bars and blue bars indicated polymorphic regions within 782-T and 782-S, 820-T and 820-S, and, 860-T and 860-S.


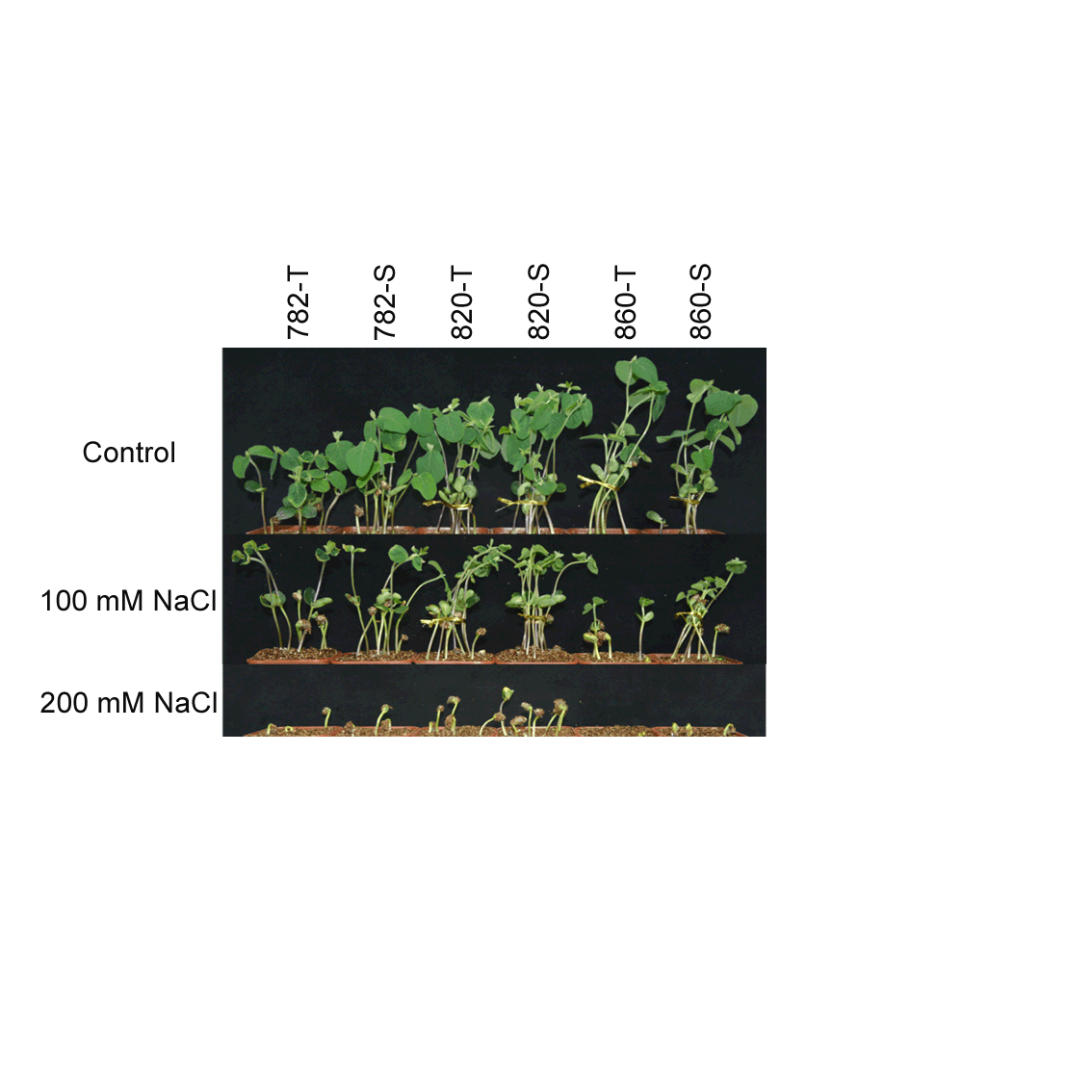


**Supplementary FIGURE 2.** Phenotype of three sets of NILs under control and 100, 200 mmol L^-1^ NaCl stress (EC = 10.6 dS m^-1^, 17.8 dS m^-1^) 15 days after sowing.

**
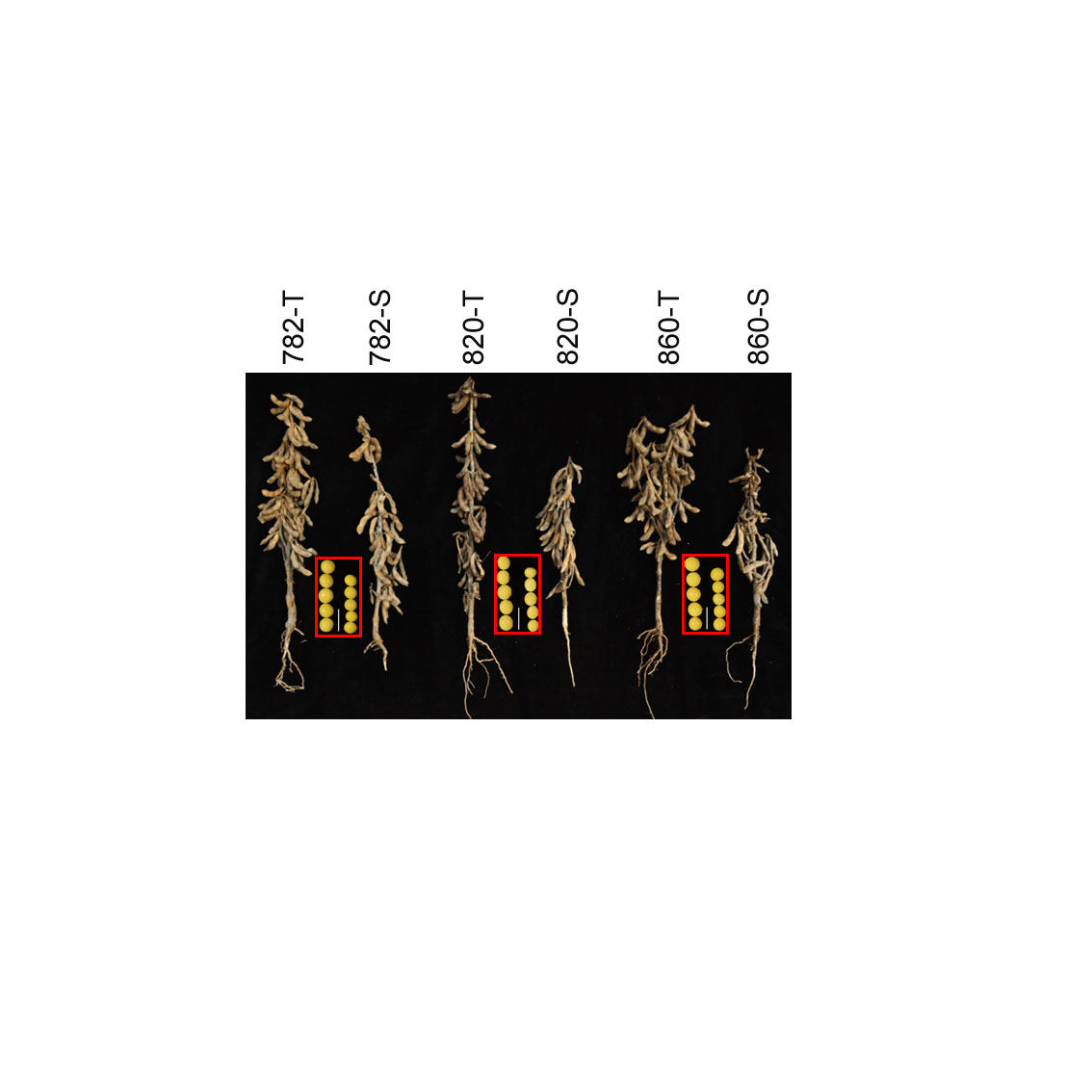
**

**Supplementary FIGURE 3.** Plant and seed phenotypes of three sets of NILs grown in saline soil (Tanghai, 2014). Image is showing plants at harvest maturity. Seeds from representative plant of each NIL are shown in red box between each pair of NIL (left for NIL-T, right for NIL-S). Scale bar for seeds, 1 cm.


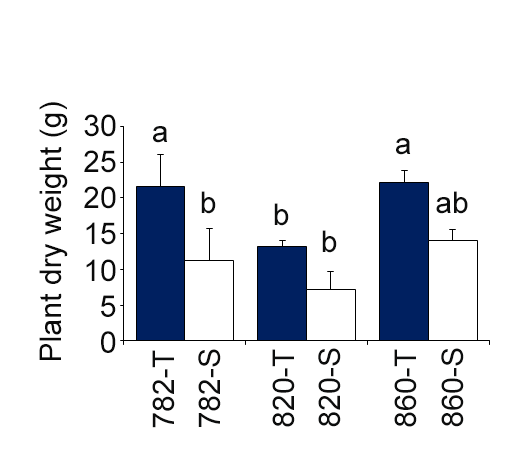


**Supplementary FIGURE 4.** Above ground dry mass of NIL lines grown on saline field at Tanghai, Hebei during 2015. Data are means of three replicates consisting of 15 bulked plants per replicate ± SD (n=3). Different letters indicate statistically significant differences between NIL lines (one-way ANOVA followed by Tukey's HSD post hoc test, P < 0.05).


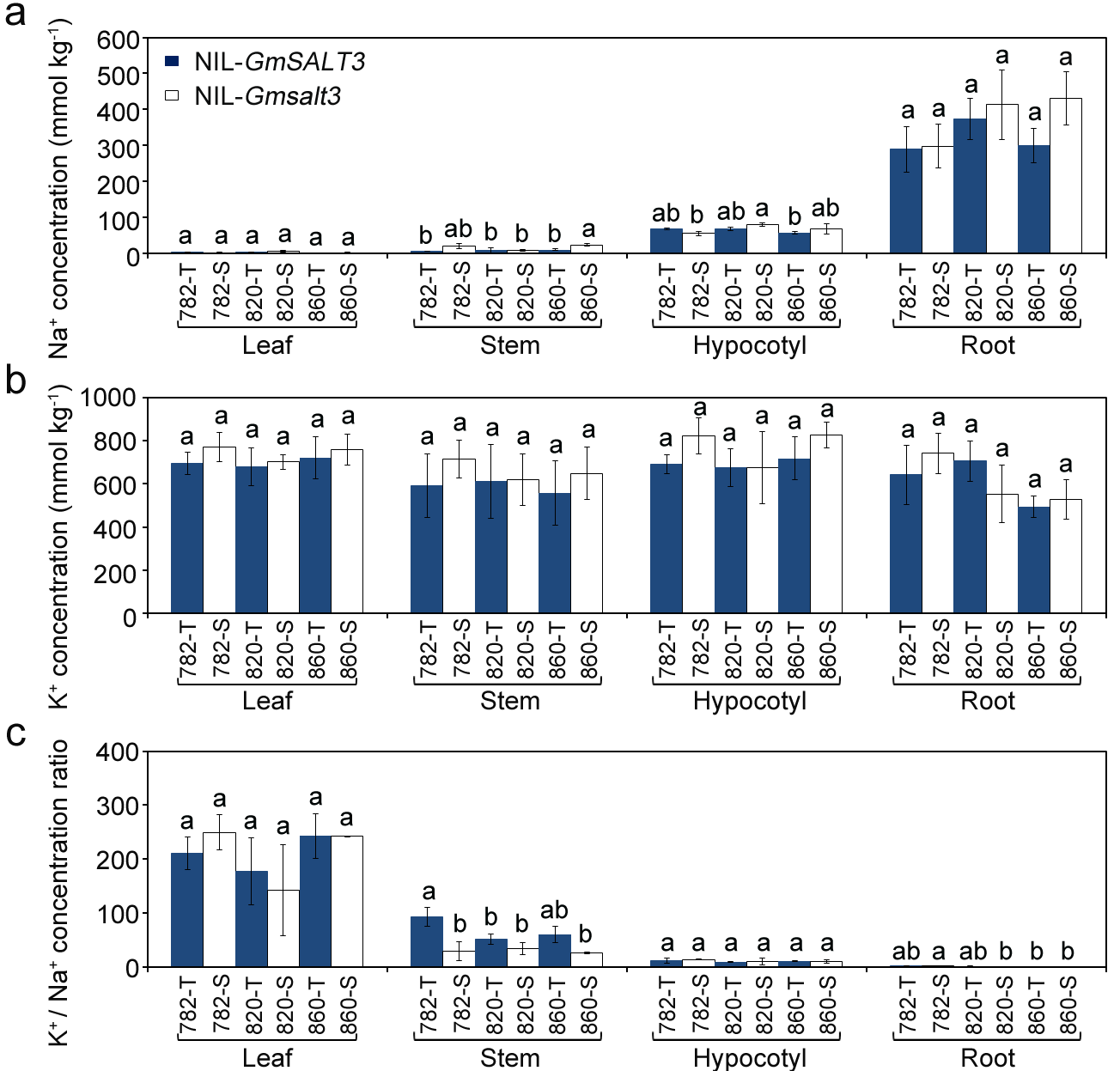


**Supplementary FIGURE 5.** Ion concentration in three sets of NILs of control treatment. **a** Concentration of Na^+^ in leaf, stem, hypocotyl and root of three sets of NILs. **b** Concentration of K^+^ in leaf, stem, hypocotyl and root of three sets of NILs. **c** The K^+^ / Na^+^ ratio in leaf, stem, hypocotyl and root of three sets of NILs. Data are means of three replicates consiting of the mean of 5 plants grown in the same pot ± SD (n=3). Different letters indicate statistically significant differences between NIL lines (one-way ANOVA followed by Tukey's HSD *post hoc* test, *P* < 0.05).
